# Improvements in Stress, Affect, and Irritability Following Brief Use of a Mindfulness-based Smartphone App: A Randomized Controlled Trial

**DOI:** 10.1007/s12671-018-0905-4

**Published:** 2018-03-01

**Authors:** Marcos Economides, Janis Martman, Megan J. Bell, Brad Sanderson

**Affiliations:** 1 Headspace Inc, 2415 Michigan Avenue, Santa Monica, Los Angeles, CA 90404 USA; 2 Department of Psychiatry and Behavioral Sciences, School of Medicine, Stanford University, 401 Quarry Road, Stanford, CA 94305 USA

**Keywords:** Mindfulness, Meditation, Well-being, Stress, Positive affect, Smartphone app, Digital health

## Abstract

Mindfulness training, which involves observing thoughts and feelings without judgment or reaction, has been shown to improve aspects of psychosocial well-being when delivered via in-person training programs such as mindfulness-based stress reduction (MBSR) and mindfulness-based cognitive therapy (MBCT). Less is known about the efficacy of digital training mediums, such as smartphone apps, which are rapidly rising in popularity. In this study, novice meditators were randomly allocated to an introductory mindfulness meditation program or to a psychoeducational audiobook control featuring an introduction to the concepts of mindfulness and meditation. The interventions were delivered via the same mindfulness app, were matched across a range of criteria, and were presented to participants as well-being programs. Affect, irritability, and two distinct components of stress were measured immediately before and after each intervention in a cohort of healthy adults. While both interventions were effective at reducing stress associated with personal vulnerability, only the mindfulness intervention had a significant positive impact on irritability, affect, and stress resulting from external pressure (between group Cohen’s *d* = 0.44, 0.47, 0.45, respectively). These results suggest that brief mindfulness training has a beneficial impact on several aspects of psychosocial well-being, and that smartphone apps are an effective delivery medium for mindfulness training.

## Introduction

There is mounting evidence that mindfulness-based interventions bring about positive physical and mental health outcomes in both healthy (Brown and Ryan 2003; Keng et al. 2011) and clinical (Hilton et al. 2017; Hofmann et al. 2010) populations. Mindfulness aims to cultivate increased moment-to-moment awareness of one’s thoughts, feelings, and bodily sensations, while maintaining an open mind, free from distraction and judgment (Kabat-Zinn 1994). A central tenet of mindfulness is that it does not aim to suppress or change direct experience, but rather the way in which present-moment experience is interpreted. By observing thoughts and feelings as mental events, rather than as reality or truths about the self, one can retrain negative thought patterns and reduce reactivity, thus fostering a greater sense of calm and well-being (Feldman et al. 2010; Hoge et al. 2014).

The most pervasive and well-established mindfulness-based interventions are mindfulness-based stress reduction (MBSR) and mindfulness-based cognitive therapy (MBCT). These 8-week long, group-based interventions involve a combination of mindfulness and other stress reduction and cognitive techniques, with up to 45 min of home practice per day. MBSR and MBCT have been shown to convey a host of psychological benefits such as reduced stress and anxiety (Khoury et al. 2015; Vøllestad et al. 2012), reduced depressive symptomatology (Hofmann et al. 2010), and increased quality of life in both cancer patients (Zhang et al. 2015) and those with chronic pain (Hilton et al. 2017). Further, these interventions have been linked to changes in executive functioning networks in the brain (Tang et al. 2012). However, factors such as cost, time demands, and the requirement to attend sessions in person may act as barriers to enrolment for a significant proportion of the population, including young professionals, those with children, those with disabilities, and those living in rural areas.

Recently, digital mediums for mindfulness training, including mindfulness-based smartphone apps, have become widely available (Plaza et al. 2013). Relative to in-person training, digital mediums are wider-reaching, demand less time, are more affordable, and may be more engaging. Digital mediums therefore have wide-reaching potential for improving public health. A recent meta-analysis of online mindfulness-based interventions has demonstrated significant beneficial impact on stress, anxiety, depression, and well-being, suggesting that digital mindfulness training may confer similar benefits to in-person training (Spijkerman et al. 2016). In support of this, several recent studies suggest that mindfulness training delivered via a smartphone app can increase quality of life (van Emmerik et al. 2017), well-being (Howells et al. 2014), and self-reported mindfulness (Plaza García et al. 2017), as well as reduce symptoms of depression (Ly et al. 2014b).

Despite this shift, research focused on the efficacy of mindfulness-based smartphone apps (which are now highly popular) remains in its infancy. An important distinction between app-based mindfulness training and structured in-person training is that the former is self-guided and thus may involve lower overall durations of practice. While some studies suggest that very short mindfulness interventions can improve psychological well-being (Creswell et al. 2014; Zeidan et al. 2011), others argue that longer interventions are required (Baer et al. 2012). In addition, previous studies of app-based mindfulness training have largely utilized inactive control groups (i.e., wait-list or no intervention) (Ly et al. 2014a; van Emmerik et al. 2017), or have focused on mindfulness as a sole outcome measure (Chittaro & Vianello 2016; Plaza García et al. 2017).

The present study aimed to assess whether completing the first 10 introductory sessions of the mindfulness-based smartphone app Headspace positively impacts stress, affect, and irritability, relative to an active control. Headspace features daily guided meditations delivered by former Buddhist monk Andy Puddicombe and was chosen due to its overwhelming popularity, with over 20 million global downloads at the time of writing. Headspace is also the highest scoring mindfulness app as per the Mobile Application Rating System (Mani et al. 2015), and has previously been shown to convey a host of benefits, including increases in mindfulness (Bennike et al. 2017; Morrison Wylde et al. 2017; Wen et al. 2017) and well-being (Howells et al. 2014). Stress was chosen as an outcome measure for several reasons. First, it is one of the most commonly reported psychological health outcomes in mindfulness research, making it easy to weigh the present results against other mindfulness interventions and meta-analyses. Second, we posited that a very brief mindfulness intervention would be more likely to impact stress than other psychosocial outcomes (such as anxiety and depression), as stress is typically associated with larger overall effect sizes (Spijkerman et al. 2016). In addition, affect and irritability were included as positive affect has previously been shown to increase following a brief in-person mindfulness intervention (Zeidan et al. 2010), and Headspace has previously been shown to reduce aggressive behavior (DeSteno et al. 2017).

A novel active control condition was utilized, which consisted of 10 excerpts from Andy Puddicombe’s audiobook *The Headspace Guide to Meditation and Mindfulness*. The sessions are narrated by Andy Puddicombe, are the same duration as the mindfulness meditation sessions, and were delivered via the Headspace app, thus closely matching the mindfulness intervention across key attributes. Specifically, the user flow through the app was identical in both the mindfulness and audiobook conditions, differing only with regards to actual session content. Compared to the active control, it was hypothesized that participants in the mindfulness intervention would demonstrate larger reductions in stress and irritability, and larger increases in positive affect.

## Method

### Participants

Participants were recruited via a third-party participant recruitment service (www.findparticipants.com). Eligibility criteria included (a) being 18–50 years old, (b) being fluent in English, (c) having not practiced any form of meditation for 6 months prior to study commencement, (d) having no prior experience with Headspace, (e) having no current or previous psychological illness, and (f) having access to a smartphone and a laptop or computer. A power analysis using the software G*Power revealed that we required 52 participants to detect a time × group interaction with 80% power (at the 0.05 significance level; Faul et al. 2007). This was based on the assumption of a small-medium effect size (Cohen’s *d* = 0.40) with respect to stress, which was in turn based on a recent meta-analysis of online mindfulness interventions (Spijkerman et al. 2016). In total, 171 volunteers from the general population expressed interest in participating. Eighty-one participants withdrew after being randomized but before beginning any of the baseline assessments or intervention sessions, and were not included in the analysis. Of the 88 (52 females, 36 males) participants that begun the intervention, 19 withdrew before completing the intervention, resulting in a final sample size of 69 (39 females, 30 males). Table 1 shows baseline characteristics of the final sample. The study was approved by the University College London (UCL) Ethics Committee (project ID 2789/001). All participants provided informed consent prior to participation.

**Table 1:** Baseline demographic characteristics for the Headspace group (*n* = 41) and the audiobook group (*n* = 28)

Study variable	Headspace (*n* = 41)	Audiobook (*n* = 28)
Age band (*n* [%])		
18–24	11 (26.9)	8 (28.6)
25–29	10 (24.4)	8 (28.6)
30–39	11 (26.9)	8 (28.6)
40–49	9 (22.0)	4 (14.3)
Gender (*n* [%])		
Male	15 (36.6)	13 (46.4)
Female	26 (63.4)	15 (53.6)
Ethnicity (*n* [%])		
White	31 (75.6)	18 (64.3)
Hispanic	2 (4.9)	1 (3.6)
African-American	1 (2.4)	0 (0)
Asian	4 (9.8)	5 (17.9)
Mixed	2 (4.9)	2 (7.14)
Other	1 (2.4)	2 (7.14)
Education (*n* [%])		
No school	0 (0)	1 (3.6)
High school	8 (19.5)	7 (25.0)
University	26 (63.4)	15 (53.6)
Post-graduate degree	7 (17.1)	5 (17.9)
Meditation has the potential to be beneficial (*n* [%])		
Strongly disagree	0 (0)	0 (0)
Disagree	4 (9.8)	1 (3.6)
Neither agree nor disagree	8 (19.5)	11 (39.3)
Agree	15 (36.6)	8 (28.6)
Strongly agree	14 (34.2)	8 (28.6)

### Procedure

The mindfulness intervention was delivered via the Headspace (www.headspace.com) mindfulness-based smartphone app, and consisted of the app’s first 10 introductory sessions, named “Take 10.” These sessions are intended to act as a general introduction to mindfulness meditation and incorporate techniques such as breath awareness and body scanning. Each session has a duration of approximately 10 min and is guided by former Buddhist monk Andy Puddicombe. The app encourages users to complete one session a day, but this was not enforced. At the time of writing, Headspace has been downloaded more than 20 million times, is the highest scoring mindfulness-based iPhone app as per the Mobile Application Rating System (Mani et al. 2015), and has previously been shown to increase compassion (Lim et al. 2015) and well-being (Howells et al. 2014), and to reduce mind wandering (Bennike et al. 2017) and aggression (DeSteno et al. 2017).

A failure to utilize rigorous control or comparison conditions has previously been discussed as a major limitation in meditation research (Davidson and Kaszniak 2015). Here, a novel active control intervention was designed (in collaboration with the Brady Reynolds lab, University of Kentucky), which consisted of 10 excerpts from Andy Puddicombe’s audiobook *The Headspace Guide to Meditation and Mindfulness*. The sessions are narrated by Andy Puddicombe, are approximately 10 min in duration, and were delivered via the Headspace app, thus closely matching the mindfulness intervention across key attributes. Specifically, the user flow through the app was identical in both the mindfulness and audiobook conditions, differing only with regards to actual session content. The audiobook sessions, being predominantly psychoeducational, introduce the background and key concepts behind mindfulness, and include reflections from Andy Puddicombe’s experience training as a Buddhist monk. The chosen excerpts excluded any content which featured guided mindfulness exercises.

Participants were recruited via an email advertisement which was delivered via a third-party recruitment service (www.findparticipants.com). The email linked participants to a screening and demographics questionnaire (see “Screening Questionnaire”), hosted by SurveyMonkey (www.surveymonkey.com). Within 24 h of completing the screening questionnaire, eligible participants were randomized, using simple randomization via a computer-generated sequence, to the experimental condition (mindfulness meditation via the Headspace app) or the active control condition (audiobook via the Headspace app), and emailed the baseline questionnaires. Alternative randomization procedures (such as blocked or stratified) were not used, as it is common in online studies for participants to withdraw after being randomized (but before beginning baseline assessments), which largely negates the benefits of such procedures. Participants were also provided with a Headspace voucher code, which restricted their access to either the experimental or control content, along with instructions on how to redeem their code and install the app. If a participant completed all 10 sessions of their intervention, they were automatically emailed the end-of-study questionnaires. Otherwise, they were considered to have withdrawn from the study. Participants were given up to 1 month to complete the intervention and did not receive any encouragement or reminder communications from the researchers. Those that completed the intervention were sent a final email thanking them for their participation, which included a £25 (or equivalent) Amazon voucher as compensation for their time. The entire experiment was run online.

### Measures

#### Screening Questionnaire

Participants responded to our study advert by completing an online questionnaire which screened for our inclusion/exclusion criteria. To be included, participants had to have access to a smartphone and a laptop or computer, be aged 18–50, be fluent in English, and have no current or previously diagnosed psychiatric illness. Participants were excluded if they had previously used Headspace or had practiced mindfulness or meditation within the past 6 months. In addition, we incorporated demographic questions including age band, gender, level of education, and ethnicity. Finally, participants were asked to rate (using a 5-point Likert scale) whether they “believe meditation has the impact to improve aspects of well-being such as stress, anxiety, or depression”.

#### Stress Overload Scale

Stress was measured using the Stress Overload Scale (SOS). The SOS is a validated 30-item scale designed to measure “stress overload,” a state described in stress theories as occurring when demands overwhelm resources (Amirkhan 2012; Amirkhan et al. 2015). The respondent uses a 5-point Likert scale (1 = not at all, 5 = a lot) to indicate subjective feelings and thoughts experienced over the prior week, which makes it particularly suitable for studies utilizing short interventions. There are two factors underlying overload: personal vulnerability and event load, which are measured by two distinct but correlated subscales. The minimum and maximum scores for each subscale are 12 and 60, respectively, with higher scores indicating higher levels of stress overload. The internal consistency of the SOS is excellent, with Cronbach’s alpha > 0.94 for both subscales and the measure as a whole. Similarly, test-retest reliability is good, with coefficients averaging 0.75 over 1 week. Construct validity has been demonstrated in community samples via significant correlations with other measures of stress and illness, and criterion validity has been shown in the SOS’ ability to predict illness following a stressful event (Amirkhan et al. 2015).

#### Scale of Positive and Negative Experience

The Scale of Positive and Negative Experience (SPANE) was used to measure levels of positive and negative affect in participants. The SPANE is a 12-item questionnaire which includes 6 items to assess positive feelings and 6 items to assess negative feelings (Diener et al. 2009). The scores are combined to create an overall affect score, which ranges from − 24 (unhappiest possible) to 24 (highest affect balance possible). The respondent uses a 5-point Likert scale (1 = very rarely or never, 5 = very often or always) to indicate how frequently they have experienced each item. The original scale asks the respondent to consider their experiences over the past month, but given the brevity of our intervention, we asked respondents to consider their experiences over the past week. The SPANE has been validated in five community samples and has demonstrated both good internal consistency (Cronbach’s alpha of 0.88 for the overall affect balance score), and test-retest reliability (with an average coefficient of 0.68 over 1 month).

#### Brief Irritability Test

The degree to which participants experienced frustration and irritability was measured using the Brief Irritability Test (BITe). The BITe is a new 5-item scale, suitable for use among both males and females, that displays minimal overlap with related constructs (Holtzman et al. 2015). The respondent uses a 6-point Likert scale (1 = never, 6 = always) to indicate how frequently they identify with each statement. The original scale asks the respondent to consider their experiences over the past 2 weeks, but given the brevity of our intervention, we asked respondents to consider their experiences over the past week. Scores across the five items are averaged to obtain a mean irritability score ranging from 1 (least irritable) to 6 (most irritable). The BITe has been validated in both healthy and patient samples and shows excellent internal consistency with Cronbach’s alpha of 0.88. Unfortunately, test-retest reliability has not yet been determined for this scale.

### Data Analyses

We performed both a complete case and an intention-to-treat (ITT) analysis. For the former, participants that failed to complete the post-intervention questionnaires were considered to have withdrawn from the study. Pearson’s chi squared was used to test for differences between group characteristics at baseline. Pre-post changes in scores between the mindfulness and audiobook control groups were compared using 2 × 2 (group × time) analysis of variance (ANOVA). Post hoc paired *t* tests were used to further characterize differences between groups. Within-group Cohen’s *d* effect sizes were calculated with (*M*_post_ − *M*_pre_)/SD_pooled_. Standard deviation was corrected for dependence by taking the correlation between pre and post score into account (SD_corrected_ = SD_pooled_ [2(1 − *ρ*)]^1/2^, where *ρ* is the correlation between scores). Between-group Cohen’s *d* effect sizes were calculated by taking the difference in score change between the Headspace and audiobook groups, dividing by the pooled pre-test standard deviation (Morris 2008).

For the ITT analysis, we fit a mixed effects model for each outcome measure, as these have the ability to handle missing data and are considered superior to other ITT approaches such as “last observation carried forward” (Baraldi and Enders 2010; Blankers et al. 2010). Predictor variables included time (coded as 1 and 2); group (coded as 1 = Headspace, 2 = audiobook); and their interaction. Each model included a random intercept across participants and was fit using maximum likelihood.

Despite being limited by a small sample size, exploratory multiple regressions were performed to assess whether baseline characteristics were predictive of changes in scores across the three questionnaires. In each case, the change in outcome score from pre- to post-intervention was the dependent variable. Explanatory variables included demographics (see “Screening Questionnaire”); participants’ beliefs about the potential benefits of meditation; time taken (in days) to complete the intervention; and intervention group (coded as 1 for mindfulness, 0 for audiobook control). All statistical tests were conducted in Matlab 2016b (www.mathworks.com) and SPSS v24, and used a statistical significance level of *p* ≤ 0.05.

## Results

### Attrition and Baseline Equivalence

**Fig. 1:**
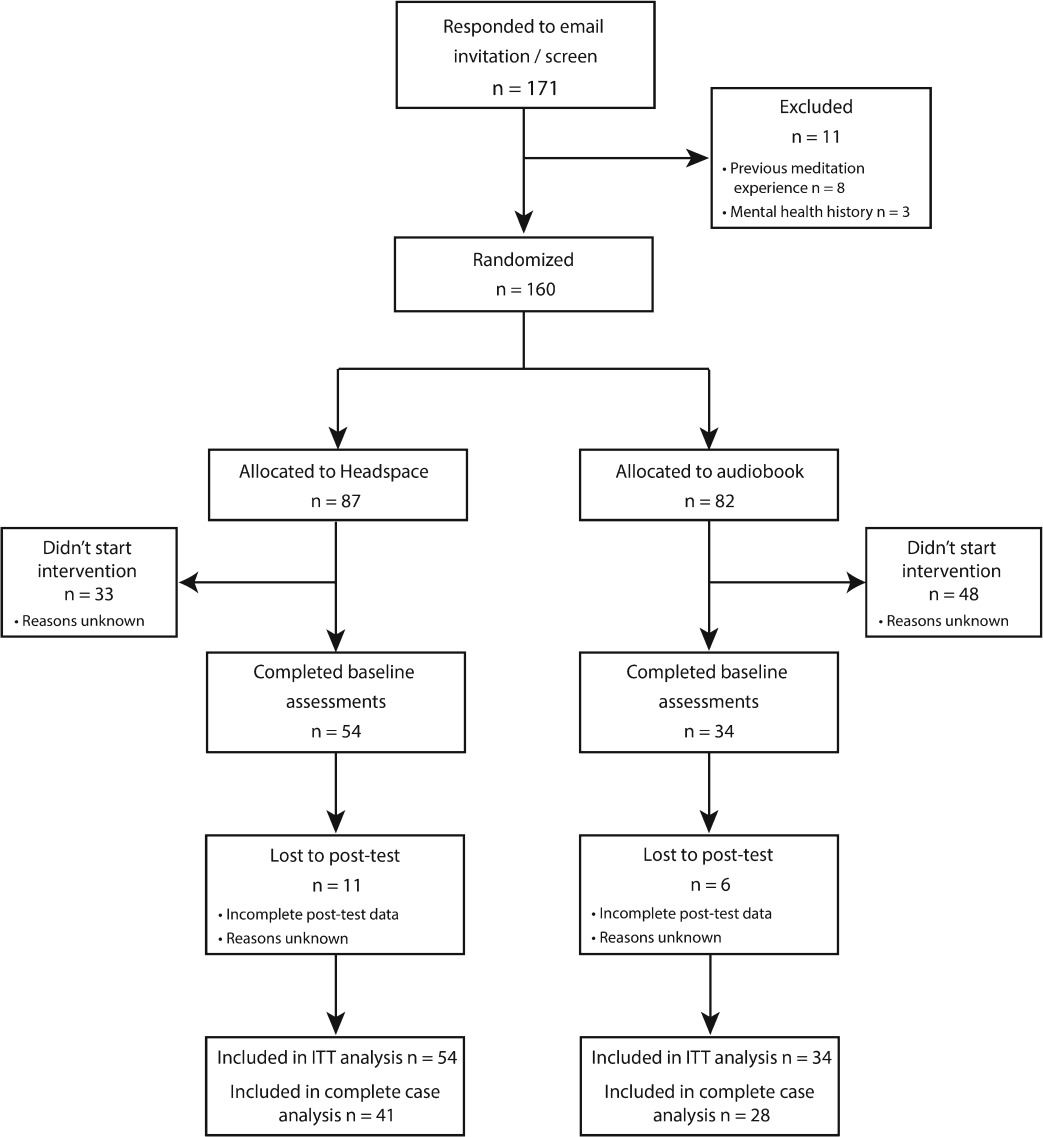
CONSORT diagram of participant flow through the study

Figure 1 displays the flow of participants through the study. Nineteen participants began the intervention but withdrew before completing the last session, resulting in a final sample size of 69 (39 females, 30 males). Of those that withdrew, 13 were from the mindfulness-meditation group, while 6 were from the audiobook group, reflecting attrition rates of 24.1 and 17.7%, respectively. Despite a slightly higher rate of attrition in the mindfulness group, the final sample sizes were 41 in the mindfulness group and 28 in the audiobook group. This disparity is predominantly due to a larger proportion of individuals in the audiobook group withdrawing after being randomized, but before beginning any of the baseline assessments or intervention sessions.

Participants in the Headspace group and audiobook group took an average of 16.2 days (SD = 6.13, min = 7, max = 30) and 15.8 days (SD = 5.16, min = 9, max = 28), respectively, to complete the intervention. Duration did not significantly differ between groups (two-sample *t* test, *p* > 0.05). Further, there were no between-group differences in demographics (*χ*^2^ test for independence, all *p* > 0.05), and no between-group differences in outcome measures (two-sample *t* tests, all *p* > 0.05) at baseline. Table 1 shows characteristics of the final sample.

### Outcomes Measures

Mean scores at baseline suggest that participants in both groups were representative of the general population with respect to stress (cf. Amirkhan 2012; Amirkhan et al. 2015), affect (cf. Diener et al. 2009), and irritability (cf. Holtzman et al. 2015). A 2 (group) × 2 (time) repeated measures ANOVA revealed a main effect of time (*F*(1,67) = 63.1, *p* < 0.001) for the personal vulnerability subscale of the SOS. However, a group × time interaction marginally failed to reach significance (*F*(1,67) = 2.94, *p* = 0.09). By contrast, the event load subscale of the SOS exhibited both a main effect of time (*F*(1,67) = 12.33, *p* < 0.001), and a group × time interaction (*F*(1,67) = 9.00, *p* = 0.004). Post hoc paired *t* tests revealed that the event load subscale scores of the SOS decreased significantly in the mindfulness group from pre- to post-intervention (*t*(40) = 4.13, *p* < 0.001), but did not change in the audiobook control group (*p* = 0.92). A between-group effect size analysis revealed a Cohen’s *d* of 0.26 and 0.45 for the personal vulnerability and event load subscales, respectively (see Table 2).

**Table 2:** Baseline and post-intervention scores (with corresponding Cohen’s *d* effect sizes) for the two subscales of the SOS, the SPANE, and the BITe in the mindfulness (Headspace; *n* = 41) group and the audiobook group (*n* = 28)

Outcome measure	Condition	Baseline mean (SD)	Day 10 mean (SD)	Cohen’s *d* (95% CI)	
				Within-group	Between-group
SOS personal vulnerability	Headspace	28.83 (11.62)	20.68 (7.75)	1.16 (0.99 to 1.96)	0.26^±^ (− 2.47 to 2.99)
	Audiobook	25.46 (11.91)	20.36 (10.92)	0.82 (0.31 to 1.40)	
SOS event load	Headspace	37.27 (12.24)	31.88 (11.85)	0.65 (0.19 to 1.08)	0.45** (− 2.44 to 3.34)
	Audiobook	33.04 (12.70)	33.14 (11.31)	0.02 (− 0.51 to 0.54)	
SPANE affect balance	Headspace	3.56 (10.59)	10.71 (8.26)	0.89 (0.36 to 1.26)	0.47** (− 1.92 to 2.87)
	Audiobook	7.18 (9.85)	9.50 (9.15)	0.37 (− 0.17 to 0.88)	
BITe	Headspace	14.39 (6.28)	10.44 (4.76)	0.80 (0.26 to 1.15)	0.44* (− 1.11 to 1.99)
	Audiobook	13.11 (7.18)	12.04 (6.51)	0.22 (− 0.29 to 0.76)	

**p ≤ 0.01; *p < 0.05; ^±^*p* = 0.09

There was a significant main effect of time (*F*(1,67) = 33.1, *p* < 0.001), and a group × time interaction (*F*(1,67) = 6.86, *p* = 0.01) for affect balance as measured by the SPANE. Post hoc paired *t* tests revealed that affect balance increased significantly in the mindfulness group (*t*(40) = 5.59, *p* < 0.001). Affect balance also increased in the audiobook control group but just failed to reach significance (*t*(27) = 1.94, *p* = 0.06). These results suggest that the mindfulness group experienced a larger increase in affect relative to baseline than the audiobook control group. The between-group Cohen’s *d* effect size was 0.47 (see Table 2). With regards to irritability, there was a significant main effect of time (*F*(1,67) = 21.3, *p* < 0.001), and a group × time interaction (*F*(1,67) = 5.51, *p* = 0.022) as measured by the BITe. Post hoc paired *t* tests revealed that irritability decreased significantly in the mindfulness group from pre-post-intervention (*t*(40) = 4.97, *p* < 0.001), but did not change in the audiobook control group (*p* = 0.25). The between-group Cohen’s *d* effect size was 0.44 (see Table 2).

To ensure that our results were not confounded by attrition, we performed an ITT analysis which included all participants with baseline data (*n* = 87). Mixed effects models revealed significant group × time interactions for the event load subscale of the SOS (*ß* = 5.18, SE = 1.79, *p* = 0.004); the SPANE (*ß* = − 4.74, SE = 1.77, *p* = 0.008); and the BITe (*ß* = 2.95, SE = 1.18, *p* = 0.013). The group × time interaction for the personal vulnerability subscale failed to reach significance (*ß* = 2.79, SE = 1.73, *p* = 0.110). Thus, the results were robust across both ITT and complete case analyses.

Lastly, we conducted exploratory multiple regressions to investigate whether any subject-specific characteristics (such as demographics and intervention duration) modulated change in score from pre- to post-intervention. However, none of the independent variables included had a significant impact on score change (all *p* > 0.05) for any of the three outcome measures.

## Discussion

A newer, more accessible form of online, self-guided mindfulness interventions have begun to show considerable promise as a means to improve psychosocial well-being (Spijkerman et al. 2016). Online mindfulness interventions have successfully been used to reduce stress in a cohort of university students (Cavanagh et al. 2013) and reduce anxiety, depression, and insomnia in a patient population (Boettcher et al. 2014). However, evidence specifically supporting the efficacy of mindfulness-based smartphone apps is scarce (Mani et al. 2015). In 2012, global smartphone shipments grew 46% to 722 million units, and there are now more than a thousand commercially available mindfulness apps. In this study, completing a brief, 10-day introductory program of a popular mindfulness app reduced average levels of self-reported stress and irritability and increased average mood ratings in our study cohort. These results were robust across both complete case and ITT analyses. Our study extends a growing body of research investigating self-help methods for increasing the dissemination of mindfulness-based interventions and suggest that mindfulness training delivered through a smartphone app has the potential to improve aspects of psychosocial well-being.

Our findings are largely consistent with previous reports that mindfulness training delivered through in-person or online formats has a beneficial impact on stress (Khoury et al. 2015), positive affect (Garland et al. 2015; Keng et al. 2011), and emotional reactivity and regulation (Britton et al. 2012; Hill and Updegraff 2012). When examining effect sizes associated with stress, recent meta-analyses have reported moderate between-group Cohen’s *d* effect sizes for both online (*d* = 0.40; Spijkerman et al. 2016) and in-person (*d* = 0.51; Gotink et al. 2015) interventions. This is consistent with a between-group effect size of *d* = 0.45 for the event load subscale of the SOS reported in the present study and suggests that mindfulness training via Headspace is at least as affective at reducing stress associated with external pressure as other digital platforms or in-person programs. Moreover, relative to several studies included in the aforementioned meta-analyses, the present study utilized a more conservative control group, and a significantly shorter intervention.

Our results also expand a small but growing body of work suggesting that app-based mindfulness training increases aspects of well-being (Howells et al. 2014; van Emmerik et al. 2017). However, most online or app-based mindfulness studies have utilized wait-list control groups. Use of inactive controls has previously been raised as a methodological limitation, as they do not account for non-specific effects of intervention engagement and can limit interpretation of study findings (Davidson and Kaszniak 2015). This was addressed in the present study by utilizing an audiobook-based active control comparison. This revealed that stress relating to personal vulnerability was effectively reduced by both the mindfulness and audiobook interventions, suggesting that this benefit was not unique to the practice of mindfulness. Since the majority of previous studies have utilized global measures of stress, it is difficult to compare this result to the existing literature. In addition, it remains unknown whether a group × time interaction would have emerged if a longer intervention period had been used. By contrast, the event load subscale of the SOS exhibited reduced scores following the mindfulness intervention, but not the audiobook control. This suggests that the ability to cope with external pressure, particularly when demand overwhelms resource, is selectively improved by mindfulness training, and may emerge as an early benefit. Previous research has attributed reductions in stress following mindfulness training to increases in dispositional mindfulness and decentering (Carmody and Baer 2007; Feldman et al. 2010), which might in turn mediate increased cognitive and behavioral flexibility, and a greater sense of goal-directedness (Carmody et al. 2009).

An important but understudied question is how much mindfulness practice is needed in order for improvements in well-being to emerge. While some studies suggest that just a single session of meditation can improve mood (Johnson et al. 2013), others have reported that measurable reductions in stress require at least 4 weeks of consistent practice (Baer et al. 2012). In the present study, just 10 sessions of Headspace were sufficient to positively impact stress, affect, and irritability. This is consistent with a previous report that 10 days of Headspace increased positive affect and reduced depressive symptoms in a cohort of “happiness seekers” (Howells et al. 2014). These results may have implications for reducing the burden of stress-related illness on economic and public health (Wiegner et al. 2015), especially when considering the low cost and accessibility of digital health platforms. However, substantial further research is needed before definitive conclusions can be drawn.

Digital delivery mediums may have the potential to address some of the methodological challenges associated with traditional interventions such as MBSR. For example, estimating the quantity or dose of mindfulness training in meditation research is often problematic (Davidson and Kaszniak 2015). This is particularly true for interventions that involve self-reported home practice or engagement with an active control that cannot be directly measured. In this study, both the mindfulness and audiobook interventions were delivered via the same smartphone app, allowing engagement to be accurately tracked, and ensuring equal dosage across conditions. A further benefit of using the same delivery medium for both interventions is the ability to match user experience, with both programs featuring the same voice and visual interface.

### Limitations and Future Research

The present study has a number of limitations. First, the decision to focus on a brief intervention period precluded investigation of how the reported effects may change over longer periods of practice. Further, no follow-up data were collected, and the extent to which participants in the mindfulness group continued to engage with meditation after the 10-day program was not measured. However, a recent study using an app-based mindfulness intervention revealed benefits that persisted for at least 3 months (van Emmerik et al. 2017).

Second, the results are limited by a modest sample size, with 69 total individuals across both intervention groups (87 for the ITT analysis). In particular, our exploratory analysis investigating modulators of score change was underpowered. Understanding who is most likely to benefit from an app-based mindfulness intervention is an important ongoing question, and future studies should attempt to replicate and expand upon our results with larger study cohorts.

Third, in an effort to keep the assessment phase as short as possible, we chose not to include a measure of mindfulness. Thus, we cannot be sure whether the benefits associated with the intervention are due to increases in mindfulness or another mechanism. However, several previous studies have reported increases in mindfulness following Headspace use (Bennike et al. 2017; Wen et al. 2017), suggesting that mindfulness likely increased in our study cohort.

Fourth, we cannot exclude the possibility that some participants in the audiobook (control) group may have sought out information regarding the Headspace app (or mindfulness in general), and subsequently engaged with meditation-related content outside of the audiobook (thus contaminating allocation to the control group). However, the potential for such contamination would likely have been reduced by the brevity of our intervention.

Fifth, participants’ scores at baseline suggest that on average, both groups were close to the general population mean on the SOS (cf. Amirkhan 2012), the SPANE (cf. Diener et al. 2009), and the BITe (cf. Holtzman et al. 2015). Thus, although previous studies suggest that mindfulness is effective at reducing symptomatology and improving well-being in patient populations (Hofmann et al. 2010; Reibel et al. 2001), caution should be applied when attempting to extrapolate the findings reported here to other cohorts. Similarly, the majority of participants in our study identified as White/Caucasian, had a university degree, and had positive expectations about the benefits of meditation prior to beginning the intervention. These characteristics substantially limit the generalizability of the results, and future studies should attempt to investigate app-based mindfulness training in more diverse study cohorts.
